# Bi_2_Se_3_/n-Si Schottky Junctions for Near-Infrared Photodetectors

**DOI:** 10.3390/nano16010067

**Published:** 2026-01-02

**Authors:** Matteo Salvato, Riccardo Ciciotti, Filippo Pierucci, Mattia Scagliotti, Matteo Rapisarda, Antonio Vecchione, Anita Guarino, Michele Crivellari, Paola Castrucci

**Affiliations:** 1 Dipartimento di Fisica and INFN, Università degli Studi di Roma “Tor Vergata”, via della Ricerca Scientifica 1, 00133 Roma, Italy; riccardo.ciciotti@roma2.infn.it (R.C.); filippo.pierucci@roma2.infn.it (F.P.); paola.castrucci@roma2.infn.it (P.C.); 2 Institute of Microelectronics and Microsystems, National Research Council (CNR-IMM), 00133 Roma, Italy; mattia.scagliotti@cnr.it (M.S.); matteo.rapisarda@cnr.it (M.R.); 3 CNR-SPIN, UOS Salerno, via Giovanni Paolo II 132, 84084 Fisciano, Italy; antonio.vecchione@spin.cnr.it (A.V.); anita.guarino@spin.cnr.it (A.G.); 4 Center for Sensors and Devices, Fondazione Bruno Kessler, 38123 Trento, Italy; crivella@fbk.eu

**Keywords:** topological insulators, Schottky junctions, thermionic field effect

## Abstract

Bi_2_Se_3_ thin films with different thicknesses are deposited on prepatterned n-Si substrates by the vapor–solid deposition method, demonstrating photodetector performances in the visible and near-infrared range up to the telecommunication wavelength 1550 nm and showing response times as low as 126 ns. The current voltage characteristics measured in the temperature range 77–300 K indicate the formation of Schottky junctions at the interface between the two materials. The nature of the junctions is discussed considering the effect of disorder at the interface induced by the Bi_2_Se_3_ film granularity. The temperature dependence of the ideality factors and the Schottky barrier heights is consistent with a thermionic field effect mechanism governing the electron motion through the interface, which is responsible for the fast response of the photodetectors.

## 1. Introduction

Infrared (IR) photodetectors have gained significant attention due to their role in a variety of applications such as military technology, remote temperature sensing, biomedical imaging, night vision, and communication [1]. However, the commercially available IR devices present some limitations due to the restricted wavelength range, high costs of fabrication, and low-temperature operation. This opens wide perspectives on the use of new materials, especially if compatible with Si technology for possible integration [2]. In the last two decades, the research focused on 2-dimensional (2D) materials as possible candidates for the substitution of Si technology thanks to their special properties, such as high charge mobility, tunable bandgap, absence of dangling bonds, and lattice structure, which is mostly of the van der Waals kind [3,4]. Particular attention was devoted to the possibility of integrating 2D materials with Si technology to resolve some important issues related to the reduction in the channel size of transistor-based devices in the post-Moore era [5,6]. In this scenario, 2D materials were widely used, confirming their usefulness in nanoelectronics [7] as well as in nanophotonics [8]. In particular, due to the possibility of tuning the electronic bandgap by changing the number of atomic layers in the lattice structure, a great plethora of 2D materials were experimented with as photodetectors covering a wide range of wavelengths from ultraviolet to far IR [8,9]. Moreover, thanks to the high optical absorbance in relation to their thickness, photodetectors based on 2D materials show figures of merit comparable to or higher than the best-performing commercial devices [10]. Despite all the excellent results reported in recent years, some problems are still present in their use in nanoelectronics, mainly related to the difficulty of obtaining large-area single-crystal samples. In most of the cases, 2D materials are obtained by the exfoliation method or as nanobelts or nanowires [11]. In all the cases, they present reduced surfaces, which, in the case of photodetector application, translates into a nanometrically large active area with the consequent difficulty of detecting the incident radiation. On the other side, the disordered surface of large-area samples reduces the electrical conductivity because of the charge scattering through the grain boundary, with a consequent increase in the time response of the photodetector [12]. These problems pushed research towards new solutions, such as, for example, the use of a material that preserves its high charge mobility despite the structural disorder of a granular surface. This is the case of Topological Insulators (TIs) [13], where the charge carriers suffer scattering only by the presence of magnetic impurities. TI represents a new state of matter where electronic surface states are topologically protected by time reversal symmetry [14]. In these materials, governed by the quantum spin Hall effect [15], electrons move in opposite directions on each surface with spin and momentum perpendicularly locked (helicity) [15]. An electronic bandgap is formed, instead, in the material bulk. For the thickness of the sample larger than a magnetic length, which in most of the cases ranges around 5 or 6 nm, electrons on the same surface can suffer backscattering only if their momentum and spin are inverted at the same time, which can happen only in the presence of a magnetic impurity. These properties give this material the unique characteristics to be used as an infrared fast photodetector where the radiation is absorbed by the bulk bandgap and the photocharges move along the high mobility surface states [13]. These characteristics are further improved when TI is interfaced with other materials, forming a rectified junction that favors the photocharge separation thanks to the interface potential [16]. The van der Waals nature of the lattice structure and the absence of dangling bonds on the surface of TI favor the growth of this material on different substrates and the formation of a heterostructure with a reduced recombination charge mechanism [17], all fundamental ingredients to investigate this material as a high-performance photodetector [18]. Among the different TIs, Bi_2_Se_3_ is one of the most studied because of its bulk electronic bandgap of 0.3 eV, which allows optical absorbance from visible to infrared [13]. Depositing Bi_2_Se_3_ on n-doped Si, a fast, wide-band photodetector can be obtained with visible light absorbed by both Si and Bi_2_Se_3_ and IR radiation absorbed only by the Bi_2_Se_3_ layer, while photocharges are delivered to the external circuit through the high mobility Bi_2_Se_3_ surface states.

In this work, we show that wideband photodetectors from visible to near IR can be obtained by using a simple and low-cost fabrication method consisting of a two-step process for depositing stoichiometric Bi_2_Se_3_ thin films on the surface of prepatterned n-doped Si substrates [19]. The obtained heterojunctions show Schottky barriers, which are responsible for photocharge separation, giving a measurable response even at *λ* = 1550 nm, where only Bi_2_Se_3_ is responsible for light absorption.

## 2. Materials and Methods

Bi_2_Se_3_ thin films with different thicknesses are deposited on n-doped Si substrates by a two-step vapor–solid deposition process [19]. In the first step, Bi_2_Se_3_ is loaded in the form of powder at the center of a quartz tube of a three-zone furnace. After pumping and flushing with Ar gas, the temperature of the central zone is raised to 590 °C in 60 min and maintained at that temperature for 2 h at the static Ar pressure of 4 mbar. During this first step, the evaporated Bi_2_Se_3_ is deposited along the inner wall of the quartz tube in correspondence with the two external zones of the furnace, which are at a lower temperature during the evaporation process. The deposited species are rich in Bi due to the high volatility of Se. At the end of this part of the process, the tube is opened, substrates are loaded along the downstream region of the tube, and an appropriate quantity of Se is added in the central zone to account for Se evaporation during the first step. Ar gas is flushed along the tube during the material and substrate loading to reduce possible oxidation of the deposited species. After closing and evacuating the tube, the temperature of all three zones of the furnace is raised to 590 °C under vacuum. All the species deposited along the inner wall of the quartz tube during the first step combine with the evaporated Se and deposit along the downstream region of the tube, where the substrates are located. With this method, we obtain up to 6 Bi_2_Se_3_ thin films whose thickness depends on the amount of material evaporated and on the distance of the substrates from the center of the furnace, with the thickest sample, labeled #1, closest to the center and the thinnest one, labeled #6, furthest from the center. Other samples grown on substrates positioned at regular distances between #1 and #6 are progressively numbered.

**Figure 1:**
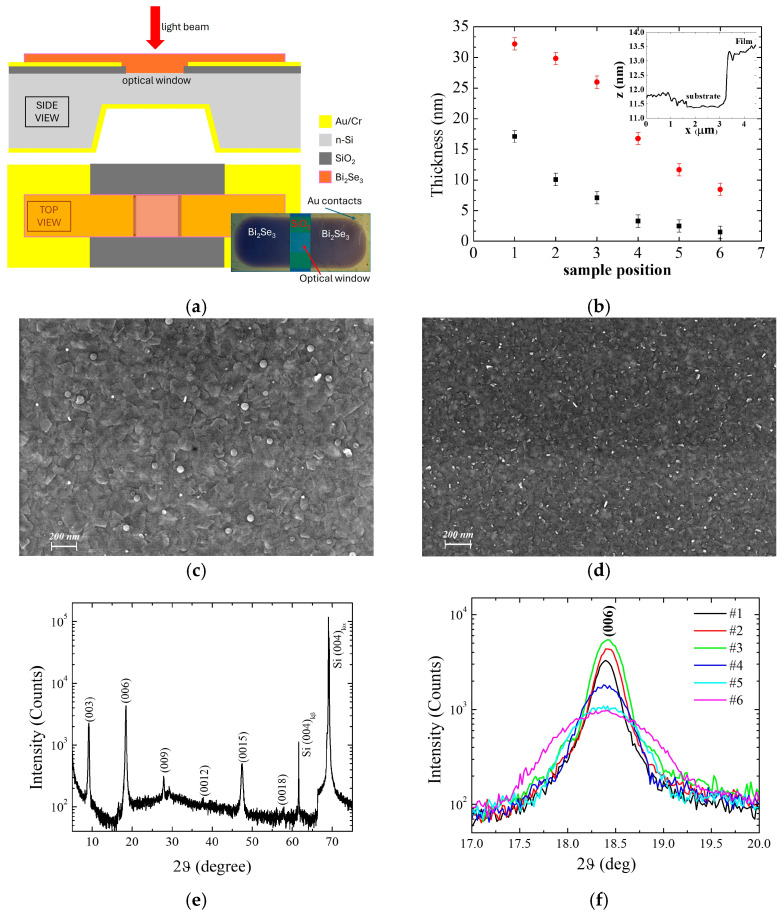
(**a**) Sketch of the photodetector. The orange layer in the side view represents the Bi_2_Se_3_ film deposited on the top surface; (**b**) Thickness of Bi_2_Se_3_ films measured by AFM (black squares) and grain dimension measured by XRD (red circles) of different films deposited on Si substrates located at position #1 to #6 from the center of the furnace; inset: AFM line profile of a Bi_2_Se_3_ film step procured for thickness measurement: (**c**) SEM image of Bi_2_Se_3_ deposited on the optical window of a substrate in position #2 and (**d**) in position #6; (**e**) XRD ϑ-2ϑ pattern of a Bi_2_Se_3_ film deposited on Si(00*l*) substrate; (**f**) XRD ϑ-2ϑ reflections of peak (006) for films grown during the same process on Si(00*l*) substrates located at positions from #1 to #6 inside the furnace.

Photodetectors were obtained by depositing a Bi_2_Se_3_ layer on the surface of an n-doped Si(001) substrate with a doping concentration of 10^16^ P-atoms/cm^3^ and with prepatterned Au contacts, realized at the Micro-Nano facility of the Fondazione Bruno Kessler (FBK) [20]. A schematic of the obtained photodetector is shown in Figure 1a, with an optical image of its surface reported in the inset. The optical window is obtained on the top side by removing, by optical lithography, an area *S* = 0.5 × 0.5 mm^2^ of 300 nm thick SiO_2_ thermally grown on the substrate surface. The metallic contacts are obtained by depositing 5 nm/150 nm of Cr/Au on the SiO_2_ patterned surface (top contact) and on the whole back surface of the n-Si substrate, which was previously overdoped to give rise to an ohmic contact (bottom contact). During the evaporation, Bi_2_Se_3_ deposits on the substrate surface, covering the n-Si optical window and the top contacts. Thanks to the metallic properties of the Bi_2_Se_3_ surface, the interface between Bi_2_Se_3_ and n-Si gives rise to a rectified Schottky junction, which is sensitive to the incident radiation. All the current-voltage (*I*–*V*) measurements were performed by voltage biasing the junctions through the top and the bottom contacts using a Keithley 2602 double-channel source meter (Tektronix, Cleveland, OH, USA). For temperature measurements, the samples were stuck on the tip of a handmade cryostat in thermal contact with a thermometer and plunged into a Dewar containing liquid nitrogen in equilibrium with its vapors. The temperature was regulated at a level of 0.1 K using a Lakeshore 330 controller (Lake Shore Cryotronics, Westerville, OH, USA). The optical measurements were performed by illuminating the optical window with visible and IR light using a *λ* = 633 nm He-Ne laser and a *λ* = 1550 nm laser diode, respectively. To evaluate the response time of the photodetector, a pulsed laser with *λ* = 450 nm is used with a pulse width of 130 ns. The morphology of all the samples was studied by investigating the Bi_2_Se_3_ surface in correspondence with the optical window using a Sigma 300 Carl Zeiss scanning electron microscope (SEM) (Carl Zeiss AG, Jena, Germany) and a Park Systems XE 100 atomic force microscope (AFM) (Park Systems Corp, Suwon, Republic of Korea). AFM was also used to measure the thickness of the Bi_2_Se_3_ films in correspondence with an artificial step obtained on appropriately deposited films on Si substrates (see inset of Figure 1b). A Bruker D2 Phaser ϑ-2ϑ x-ray diffractometer (XRD) (Bruker Corporation, Karlsruhe, Germany) was used for crystal structure characterization. Due to the limitation in the XRD detector sensitivity, thicker samples with respect to those deposited on the optical windows for photodetector applications were appropriately grown for this purpose on Si(001) substrates.

## 3. Results and Discussion

### 3.1. Structural Properties

The thickness of the samples, as measured by AFM in correspondence with an artificial step between the Bi_2_Se_3_ film and Si substrate, is shown in Figure 1b (black closed squares) as a function of the sample position inside the tube furnace. The thickness decreases going from sample #1, positioned closest to the material source, to sample #6, positioned furthest from the source. The enhanced non-linearity observed for the thinnest films is attributable to the interplay between the artificial step and the intrinsic surface corrugation associated with the grain morphology (as is evidenced from the step profile reported in the inset of the same figure for one of the measured samples), which affects the measurement resolution and the reliable extraction of the very small step heights. Figure 1c,d show the SEM images acquired in correspondence with the optical windows of samples #2 and #6, respectively. For both the samples, the surface shows a compact structure made of flat grains whose size increases with the film thickness, going from an average area of 6 × 10^3^ nm^2^ for sample #6 to 2 × 10^4^ nm^2^ for sample #2. The surface of such grains appears to be parallel to the substrate surface, albeit randomly tilted around the perpendicular direction. For XRD measurements, a different series of thicker Bi_2_Se_3_ films was appropriately deposited to allow for reasonable counts at the diffractometer detector. Figure 1e shows the XRD ϑ-2ϑ spectrum of one of the obtained films with the (00*l*) peaks corresponding to the R-3m structure of Bi_2_Se_3_. The substantial absence of other peaks, apart from the Si(004) substrate reflection, confirms the single phase of the sample and its high orientation with the *c*-axis perpendicular to the substrate surface. The *c*-axis length resulted in 2.86 nm, in agreement with the expected values for this structure [21]. Similar spectra are observed for all the samples, and in Figure 1f are shown the (006) reflections acquired for all of them. By measuring the position and the full width at half maximum of each peak, an estimation of the thickness of the grains along the *c*-axis direction is obtained by the Debye-Sherrer relation [22], and their value is reported in Figure 1b (red closed circles) as a function of the sample position inside the tube furnace. The grain thickness decreases almost linearly with the sample position, in agreement with the AFM thickness measurements, showing a decrease in the grain size with the thickness of the film. A comparison with SEM images indicates that the thicker the films, the bigger the grains, as expected for a three-dimensional coalescence mechanism.

### 3.2. Charge Transport Mechanism

**Figure 2:**
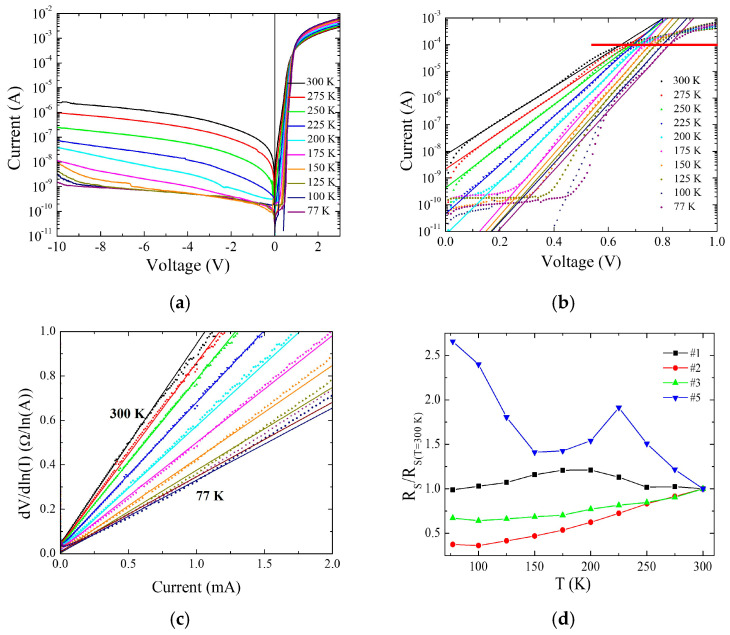
(**a**) *I*–*V* characteristics of sample #2 at different temperatures; (**b**) same as (**a**) but for positive bias. The straight lines are fits to the data for *I*_0_ and *n* determination; (**c**) *dV*/*dln*(*I*) vs. *I* for sample #2. The straight lines are linear fits to the data for *R_S_* determination; (**d**) normalized series resistance with respect to their value at room temperature for samples #1, #2, #3, and #5.

Figure 2a shows *I*–*V* characteristics, in semilogarithmic scale, of sample #2 measured in the bias voltage range −10 V to +3 V at different temperatures. A rectification ratio of 10^4^ at ±2 V is measured at *T* = 300 K, demonstrating the achieved rectification properties of the Bi_2_Se_3_/n-Si heterojunctions. Reducing the temperature down to *T* = 77 K, the rectification ratio increases up to 2 × 10^7^ indicating a strong temperature dependence of the saturation current. The positively biased part of the *I*–*V* characteristics is shown in Figure 2b. The experimental data show exponential behavior (linear in semilogarithmic scale) in a voltage range that depends on the temperature. Above this range (for current above the red line in the figure), all the curves tend to become flattened, indicating a deviation from the exponential law. This is due to the presence of a series resistance, which is taken into account in the theory by the term *I*∙*R_S_* in the expression [23]

I = I_0_e^(q(V-IR^_S_^)/nk^_B_^T)^ (1)

where *I*_0_ is the saturation current at *V* = 0, *q* is the electron charge, *n* is the ideality factor, *k_B_* is the Boltzmann constant, and *R_S_* is the series resistance. This expression, unresolvable analytically, can be modified in the form:

dV/dlnI = IR_S_ + nk_B_T/q (2)

for extracting the *R_S_* parameter by the linear fit of the data. Figure 2c shows the procedure for sample #2 at all the measured temperatures. The obtained *R_S_* values at *T* = 300 K are reported in Table 1 for all the measured samples. Due to the reduced thickness of the films, in some cases, electrical contacts were not stable, and measurements failed, as in the case of samples #4 and #6. The room temperature value of *Rs* is of the same order of magnitude for all the samples except for the thinnest ones, which show a huge increase. Since the Si contribution is the same for all the samples, we can ascribe, with good approximation, the difference between *Rs* to the Bi_2_Se_3_ and, in particular, to the film thickness. Figure 2d shows the temperature dependence of the normalized series resistance with respect to its value at *T* = 300 K (*Rs***_(*T*=300 K)_**) for all the samples.

As is evident from the figure, the samples with the two intermediate thicknesses (#2 and #3) show a metallic behavior with *Rs* that decreases with the temperature. Going to the thicker sample (#1), a semiconducting to metallic transition is observed at about 200 K, whereas a very different behavior is shown in the *Rs* vs. *T* dependence of the thinnest sample (#5).

The data in Figure 2d indicate that samples #2 and #3 have a consistent metallic component at all temperatures, in agreement with the expected topological behavior where metallic surface states develop. On the contrary, sample #1 shows metallic behavior only below 150 K, probably due to a competition between metallic surface and semiconducting bulk states whose contribution is frozen out at low temperature because of the activation mechanism of the conduction band population [24]. Finally, the thinnest sample shows a semiconducting behavior at low temperature, which is remarkable in granular samples when charge carriers remain confined inside grains when the thermal activation mechanism is reduced [25].

**Figure 3:**
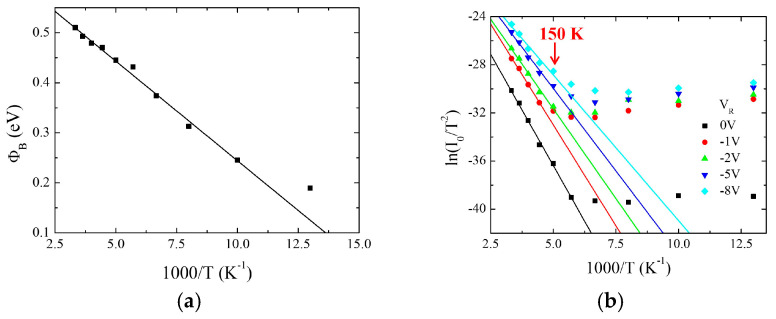

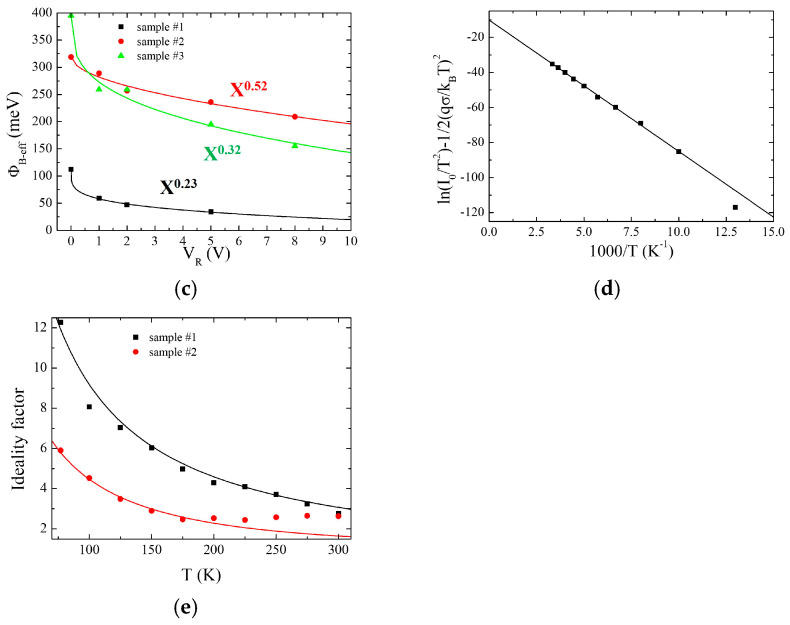
(**a**) Apparent Schottky barrier height vs. 1/T for sample #2. The line is a linear fit to the data using Equation (4); (**b**) Richardson plots for different reverse biases for sample #2. The lines are fit to the data for *T* ≥ 150 K for effective Schottky barrier height and Richardson constant determination; (**c**) *Φ_B-eff_* as determined by Richardson plots of Figure 3b vs. reverse voltage *V_R_*. The lines are fit to the data using a power law expression; (**d**) modified Richardson plot for A** calculation; (**e**) ideality factor vs. T for samples #1 (black squares) and #2 (red circles). The lines are fit to the data using expression (6).

For low current (below the red line in Figure 2b), the *Rs* contribution becomes negligible, and the *I*–*V* data are well represented by Equation (1) with *I*∙*Rs* = 0 [23]. This is evident in Figure 2b, where the *I*–*V* characteristics show a wide linear range. In this range, whose width is temperature dependent, the fit to the data allows us to estimate the values of *I*_0_ and *n* for all the measured temperatures. The obtained values of *I*_0_ give the temperature dependence of the apparent Schottky barrier height *Φ_B_* by the expression:

I_0_ = S∙A*T^2^e^−(qΦ^_B_^/k^_B_^T)^ (3)

obtained by thermionic theory based on metal/semiconducting (M/S) barriers [26]. Here, A* = 112 A/cm^2^K^2^ [26] is the ideal Richardson constant for Si. The *Φ_B_* vs. 1/*T* data are reported in Figure 3a for sample #2. It is worth noting here that *Φ_B_*, as calculated by Equation (3), represents the zero-voltage barrier height and that its dependence on the temperature is related to the *I*_0_ dependence being no explicit expression of *Φ_B_* vs. *T* in Equation (3). As shown in Figure 3a, *Φ_B_* increases with the temperature as expected because of the increase in *I*_0_ due to the thermionic effect. The effective Schottky barrier height, *Φ_B-eff_*, is obtained by the slope of the ln(*I*_0_/*T*^2^) vs. 1/*T* data plotted in Richardson fashion as shown in Figure 3b for different values of the reverse bias voltage *V_R_* [23]. The linear dependence of the data acquired at *V_R_* = 0 V for *T* ≥ 150 K is in agreement with Equation (3) and demonstrates the M/S nature of the Bi_2_Se_3_/n-Si heterojunction and the thermionic effect as responsible for the transport mechanism above this temperature. Reducing the temperature, the experimental data deviates from linearity, suggesting the occurrence of different phenomena [27]. Similar behavior is shown at all measured *V_R_* values. The voltage dependence of *Φ_B-eff_* as extracted from the slopes of the different curves is reported as a function of |*V_R_*| in Figure 3c. For all the examined samples, *Φ_B-eff_* decreases with the increase of *V_R_*, a behavior that is typical of an unpinned Fermi level at the semiconductor surface [27]. The experimental data follow a power law dependence whose exponents range between 0.25 and 0.5 for samples #1, #2, and #3, in agreement with that expected in the case of image force lowering [28] and the presence of strip-like inhomogeneity [29]. The Richardson constants A* as obtained by the fit to the data at *V_R_* = 0 V in Figure 3b are in the range 10^−6^–10^−4^ A/cm^2^K^2^ for all samples. It is worth noting that using other theories [30] based on Landauer formalism for *I*_0_ vs. *T* dependence and adopted for 2D materials, the values of A*, obtained with the same procedure, remain confined in the same range. These values are extremely smaller than the ideal one but in agreement with those obtained by other authors using this method, confirming that a different procedure for its determination is required [23,31]. 

The ideality factors for all the samples calculated at *T* = 300 K are reported in Table 1. All the obtained *n* values are between 2 and 3 except for the thinnest sample (#5), for which *n* = 5.8. The values *n* > 1 can be ascribed to different effects such as charge recombination, charge image, or interface inhomogeneity. Among them, interface inhomogeneity seems to be the most relevant in our samples, where, according to SEM analysis, a granular structure is observed in all of them. The presence of such grains affects the current through the interface because of possible different contacts with the Si surface. Therefore, instead of a uniform current through the Bi_2_Se_3_/Si interface, a current distribution is expected whose spread depends on the number of grains and the kind of contact between Bi_2_Se_3_ grains and the Si surface. Such contact can be different from grain to grain because of their orientation, their size, and possible oxide formation. These favor charge recombination and can be considered as the main cause of the non-ideality of the heterojunctions, as confirmed by the highest *n* value obtained for the thinnest film, which presents the least uniform morphology. According to Equation (3), the current distribution can be viewed as regulated by a distribution in the parameters present in the equation and, mainly, by the Schottky barrier height. Assuming a Gaussian distribution of the barrier heights, an estimation of the average value *Φ_Bm_* can be obtained by the expression [32]:

Φ_B_ = Φ_Bm_ − qσ^2^/2k_B_T (4)

where *σ* is the standard deviation. Both *Φ_Bm_* and *σ* can be obtained by the linear fit of *Φ_B_*, as extracted by Equation (3), vs. 1/T data as reported in Figure 3a for sample #2. Similar behavior is shown by all other samples. Using these values of *Φ_Bm_*, the work function *Φ_M_* of Bi_2_Se_3_ has been calculated considering the Mott–Schottky relationship [26,33], *Φ_M_* = *Φ*_*Bm*_ − *χ_Si_*, where *χ_Si_* = 4.05 eV is the electron affinity of Si. The obtained values are reported in Table 1. For all the samples, *Φ_M_* is in the range 4.7–4.9 eV, in good agreement with data reported in the literature [34] and with that obtained for samples grown with the same method and measured by the Kelvin probe technique [35]. The uncertainty *σ* on *Φ_Bm_* allows us to give a better estimation of the ideal Richardson constant A*. By combining Equations (3) and (4), one obtains [31]:

ln(I_0_/T^2^) − 0.5(qσ/k_B_T)^2^ = ln(SA*) − qΦ_Bm_/k_B_T (5)

The first member as a function of 1/*T* is shown in Figure 3d for sample #2. The linear dependence in the whole temperature range, in contrast with the Richardson plot of Figure 3b, suggests that the method works, indicating in the presence of disorder the main cause of the non-linearity of the Richardson plot below *T* = 150 K. By fitting the data, a new value of the parameter A* is obtained and reported in Table 1 as A**. The obtained values are much higher than A*, even though not comparable to that expected for Si. Considering that the thermionic emission theory gives A* = 4πqm*k_B_^2^/*h*^3^ with m* the electron mass, the low value with respect to Si can be due to the reduced value of the effective electron masses at the Bi_2_Se_3_/Si interface, as expected for surface topological states [36]. Other possible causes can be due to the presence of different mechanisms of transport than simple thermionic emission, whose effect combines with barrier inhomogeneity, as will be suggested below.

Figure 3e shows the temperature dependence of the ideality factor *n* for samples #1 and #2. In the case of the metallic sample #2, the ideality factor is almost constant for *T* > 175 K, while its value rises at low temperatures, indicating that some phenomena different from the thermionic effect are acting. This is further suggested by considering the *I*–*V* characteristics of the same sample reported in Figure 2b for *T* < 200 K, where a deviation from the linear behavior is observed at low voltage, as well as by the Richardson plot at *V_R_* = 0 in Figure 3b, where the linear dependence is not observed anymore for *T* < 150 K. At these low temperatures, the number of charges passing through the Bi_2_Se_3_/n-Si interface is strongly reduced, and an electric field is necessary to activate the conduction. The solid curves in Figure 3e are fits to the *n* vs. *T* data for samples #1 and #2 using the expression [37]:

n = (E_00_/k_B_T)coth(E_00_/k_B_T) (6)

where *E*_00_ = *h*/4*π*(*N_D_*/m*ε_s_)^1/2^ is the barrier height for thermionic field emission. Here *h* is the Planck constant, m* is the electron mass, and *ε_s_* is the dielectric constant. In the case of sample #2, the data are well fitted for *T* ≤ 175 K, giving *E*_00_ = 38 meV, indicating that an electric field-assisted mechanism of transport is activated at low temperature when the thermal energy *k_B_T* is almost halved compared to its value at room temperature. The same behavior is observed for the other metallic sample #3, while for the thickest sample (#1), the *n* vs. *T* data is well approximated by Equation (6) in the full temperature range with *E*_00_ = 79 meV. In this case, the thermal energy *k_B_T* at the considered temperature (up to 300 K) is below 1/3 of *E*_00_, and the addition of the electric field is necessary for overcoming the Schottky barrier height in agreement with the thermionic field effect model. From the obtained values of *E*_00_, the calculation of the effective electron masses gives *m_e_* = 2 × 10^−4^ ∙*m_0_* and *m_e_* =4.8 × 10^−5^∙*m*_0_, with *m*_0_ the free electron mass, for samples #2 and #1, respectively. The calculated electron masses appear to be much lower than expected in the case of Si, confirming the presence of Dirac states at the Bi_2_Se_3_/n-Si interface, which are characterized by massless charge particles.

### 3.3. Optical Properties

**Figure 4:**
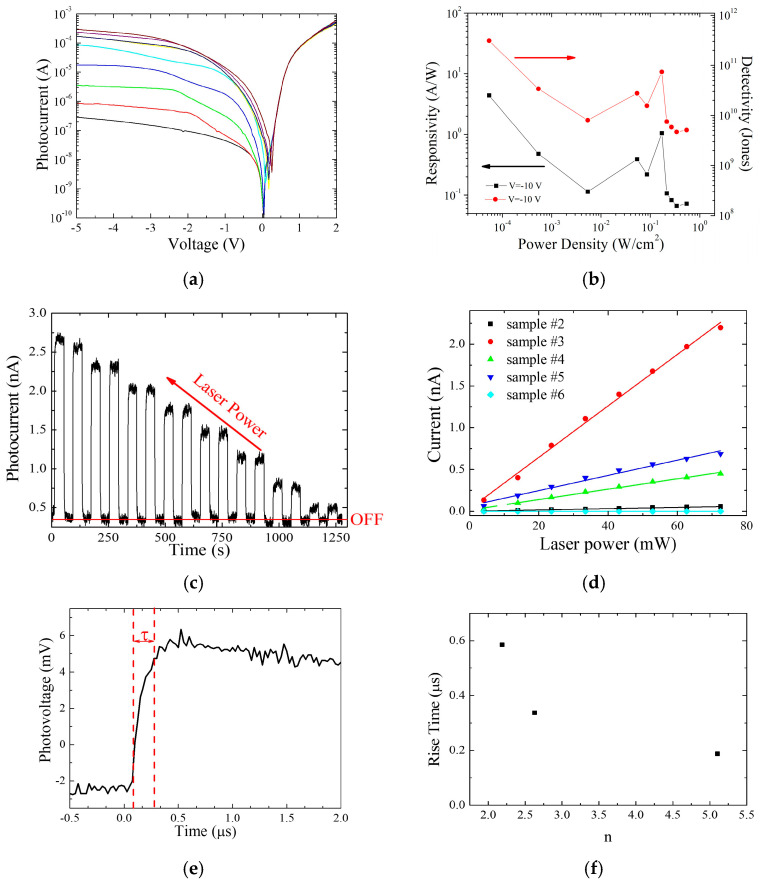
(**a**) *I*–*V* characteristics of Bi_2_Se_3_/n-Si photodetector illuminated with visible light (*λ* = 633 nm) and at different light powers; (**b**) responsivity and detectivity of the same sample of (**a**) as a function of light power density; (**c**) photocurrent response at zero bias voltage of the same sample illuminated with IR light at *λ* = 1550 nm and at different light powers; (**d**) photocurrent vs. light power at *λ* = 1550 nm for samples with different Bi_2_Se_3_ thickness; (**e**) time response of the same film under visible light pulse at *λ* = 450 nm; (**f**) rise time vs. ideality factor for three different photodetectors.

As expected, the heterojunctions show a response to incident light. Figure 4a reports typical *I*–*V* characteristics at *λ* = 633 nm for different powers of the incident light acquired for sample #3. The inverse current increases with the laser power up to a saturation value where the curves overlap. The best result obtained in terms of the ratio between the light and dark conditions is 2.1 × 10^3^ at *V_R_* = −5 V. The optical characteristics of the devices are reported in Table 2, while Table 3 lists the most common parameters reported by the literature for some recent TI/Si heterojunctions. The dark current measured at *V_R_* = −10 V in our samples decreases with the Bi_2_Se_3_ film thickness, as expected if one considers that the current is transferred to the external circuit through the Bi_2_Se_3_ layer. Responsivity and specific detectivity for all the samples are calculated using the expressions *R* = (*I_light_* − *I_dark_*)/*P_light_* and D* = *R*·(*S*/2*qI_dark_*)^1/2^, respectively [38]. Figure 4b shows the power density dependence of *R* and D* for sample #2. A responsivity of 4.5 A/W at *V_R_* = −10 V and a detectivity of 3.1 × 10^11^ Jones at the same voltage are measured for this sample. All the other samples give light/dark ratio values of the order of 10^3^ and responsivity greater than 0.1 A/W, apart from sample #6, which is mostly granular, as already observed. Interestingly, all the samples respond to IR light at *λ* = 1550 nm. Figure S1 in the Supplementary Material shows the absorption spectra of Bi_2_Se_3_ thin films with different thicknesses deposited with the same technique on a transparent substrate. Although the main absorption is in the visible, a consistent signal is also detected at IR wavelengths. Figure 4c shows the light-on, light-off response of sample #3, which gives a photocurrent as high as 2.7 nA under a radiation power of 72 mW. Although the difference in the photoresponse between visible and IR radiation is high, it must be considered that in the case of *λ* = 633 nm, the radiation is absorbed by both silicon and Bi_2_Se_3_, while in the case of *λ* = 1550 nm, only Bi_2_Se_3_ participates in the production of photocharges, silicon being transparent at this wavelength. 

The possible presence of intraband levels due to interface defects or oxygen doping gives a negligible contribution, as confirmed by photocurrent measurements, performed at IR wavelengths in the range 1000–2600 nm. Therefore, the photocharges are generated inside the Bi_2_Se_3_ layer, whose thickness ranges between 3 nm and 30 nm for all the considered samples. For all of them, the photoresponse at *λ* = 1550 nm is a linear function of the incident power, as reported in Figure 4d. The difference in the response between the samples can be ascribed to a combination of Bi_2_Se_3_ film thickness, its metallic properties, and Schottky barrier height. The Schottky nature of the heterojunction has important consequences on the speed of the response of the devices to the illuminating radiation. Figure 4e shows the photodetector response to a light impulse at *λ* = 450 nm. 

The rise time *τ*, measured as the time interval between 10% and 90% of the maximum photocurrent, is in the range of 120–350 ns for all the investigated samples. Such a fast response is uncommon in TI/Si heterojunctions, which normally show response times in the range 100 μs–10 ms [26] as reported in Table 3. Such high speed, also considering the limited width of the pulsed laser beam of 130 ns, can be attributed to a double mechanism of photocharges separation by interface potential and high mobility in the Bi_2_Se_3_ layer. The field effect-enhanced thermionic mechanism is responsible for the high velocity of the photocharge separation at the interface. These photocharges are then carried towards the external circuit through the high mobility Dirac states of Bi_2_Se_3_, contributing to the fast response of the photodetector. This is confirmed by the data reported in Figure 4e, showing the dependence of the rise time on the ideality factor. As discussed above, a high ideality factor is consistent with non-ideality of the Schottky barrier, which, in our case, is characterized by the thermionic field effect mechanism. The presence of an electric field, which adds to the thermal effect in favoring the barrier crossing of the charges, enhances the speed of the detector, reducing the response time.

## 4. Conclusions

Bi_2_Se_3_/n-Si heterojunctions with different Bi_2_Se_3_ thickness layers are successfully fabricated by vapor–solid deposition. The *I*–*V* measurements at different temperatures show that a Schottky barrier is achieved and that charge transport through the barrier happens by a thermionic mechanism enhanced by the presence of the intrinsic electric field. Optical measurements demonstrate the response of these devices at visible and IR wavelengths up to *λ* = 1550 nm, suitable for telecommunication applications. The role of Bi_2_Se_3_ as a topological insulator is evidenced by the high speed of the devices at the light impulse, where high-mobility surface states, enhanced by the thermionic field effect mechanism, allowed us to obtain responses as fast as hundreds of nanoseconds.

## Figures and Tables

**Table 1:** Data extracted from *I*–*V* measurements at different temperatures. *R_S_* and *n* are at *T* = 300 K. Electrical measurements were not performed for samples #4 and #6 due to contact instability.

Sample	Thickness (nm)	*R*_*S*(*T*=300 K)_ (kΩ)	*n*	*Φ_M_* (eV)	A** A/cm^2^K^2^	*E*_00_ (meV)
1	17.1	5.6	2.8	4.90	0.03	79
2	10.1	1.9	2.6	4.69	0.02	38
3	7.1	10	2.2	4.83	0.1	47
4	3.3	-	-	-	-	-
5	2.5	59	5.8	4.74	0.5	-
6	1.5	>100	-	-	-	-

**Table 2:** Optical properties of the photodetectors. Responsivity and detectivity are calculated at *V_R_* = −10 V.

Sample	*I_dark_* (A)	*R* (A/W)	D* (Jones)	Rise Time (ns)
2	1.4 × 10^−5^	4.5	3.1 × 10^11^	337
3	1.1 × 10^−6^	0.53	1.4 × 10^11^	-
4	3.1 × 10^−7^	0.13	7.1 × 10^10^	126
5	2 × 10^−7^	0.24	1.5 × 10^11^	188
6	2 × 10^−7^	0.06	3.6 × 10^10^	-

**Table 3:** Comparison with the performances of some relevant TI/Si photodetectors.

Material	Response Time (μs)	λ nm	R (A/W)	D* (Jones)	Processing Temperature (°C)	Structure	Ref
Bi_2_Se_3_/Si	19.7	980	7.6	6.3 × 10^12^	374	film	[39]
Bi_2_Te_3_/Si	0.3 × 10^6^	635	8.9	2 × 10^9^		film	[40]
Sb_2_Te_3_/Si	130 × 10^3^	2400	270	1.3 × 10^13^	300	film	[41]
TlBiSe_2_/Si	-	900	52	1.6 × 10^12^	-	film	[42]
Bi_2_Te_2_Se/Si	458 × 10^3^	650	19.6	8 × 10^11^	230	film	[43]
SnTe/Si	8		3.7	8.4 × 10^12^		film	[44]
Bi_2_Se_3_/Si	520 560	635 1550	7.2 × 10^−3^ 3 × 10^−5^	1.2 × 10^11^ 1.4 × 10^5^	250	Pyramidal Si	[12]
Na-Sb_2_Se_3_/Si	13.6 × 10^3^	532	4.3	2.5 × 10^9^		film	[45]
Bi_2_Se_3_/Si	45 × 10^3^	808	10^3^	3 × 10^12^		nanowire	[46]
Bi_2_Se_3_/Si	2.5		24.3	4.4 × 10^12^	-	film	[47]
SnTe/Si	0.2 × 10^6^	254	71.1	-		nanoflakes	[48]
Bi_2_Se_3_/Si	0.126	633	4.5	3.1 × 10^11^	<100	film	This work

## Data Availability

The original contributions presented in this study are included in the article/Supplementary Material. Further inquiries can be directed to the corresponding author.

## Supplementary Materials

The following supporting information can be downloaded at: https://www.mdpi.com/article/10.3390/nano16010067/s1. Figure S1 shows the absorbance spectra obtained for Bi_2_Se_3_ thin films deposited on quantz substrate by the same method used for Bi_2_Se_3_/n-Si junctions cited in the article. The spectra show absorbance high absorbance in the visible range but a relevant absorbance also above 1000 nm confirming the possibility to use Bi_2_Se_3_ films as infra-red detectors. The sudden change in the curves at *l* = 850 nm is due to a change of detector in the experimental equipment.

## Abbreviations

The following abbreviations are used in this manuscript:

IR	Infrared
XRD	X-ray diffraction
SEM	Scanning electron microscope
AFM	Atomic force microscopy
M/S	Metal/semiconducting
