# Supplementary material for: Bi2Se3/n-Si Schottky Junctions for Near-Infrared Photodetectors
Source: Nanomaterials (Basel). 2026 Jan 2;16(1):67. doi: 10.3390/nano16010067 (PMC12787348; doi:10.3390/nano16010067)
Supplement: Supplementary file 1 [file nanomaterials-16-00067-s001.zip › nanomaterials-4067921-supplementary.pdf]

Bi2Se3/n-Si Schottky junctions for near infrared photodetectors

Supplementary Materials

Matteo Salvato[1,*], Riccardo Ciciotti[1], Filippo Pierucci[1], Mattia Scagliotti[2], Matteo Rapisarda[2], Antonio Vecchione[3], Anita Guarino[3], Michele Crivellari[4], and Paola Castrucci[1]

[1]*Dipartimento di Fisica and INFN, Università degli Studi di Roma "Tor Vergata", via della Ricerca Scientifica 1, 00133 Roma-Italy*

[2]*Institute of Microelectronics and Microsystems, National Research Council (CNR-IMM), 00133 Roma, Italy*

[3]*CNR-SPIN, UOS Salerno, via Giovanni Paolo II 132, I84084 Fisciano, Italy*

[4]*Center for Sensors and Devices, Fondazione Bruno Kessler, 38123 Trento, Italy*

[*]*Correspondence: matteo.salvato@roma2.infn.it; Tel.: +390672594506*

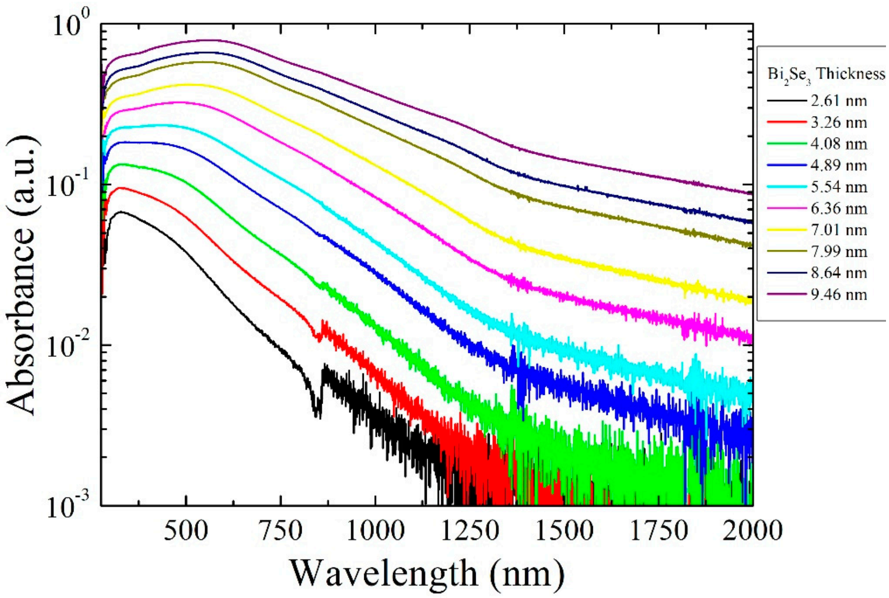

Figure S1. Absorbance spectra of Bi_2Se_3 thin films with different thickness.

Figure S1 shows the absorbance spectra obtained for Bi_2Se_3 thin films deposited on quartz substrate by the same method used for Bi_2Se_3/n-Si junctions cited in the article. The spectra show high absorbance in the visible range and relevant absorbance above 1000 nm confirming the possible use of Bi_2Se_3 films as infra-red detectors. The sudden change in the curves at λ=850 nm is due to a change of detector in the experimental equipment.
